# Independent Factors Affecting Postoperative Short-Term Urinary Continence Recovery after Robot-Assisted Radical Prostatectomy

**DOI:** 10.1155/2021/9523442

**Published:** 2021-11-28

**Authors:** Wen Deng, Ru Chen, Xian Jiang, Ping Zheng, Ke Zhu, Xiaochen Zhou, Xiaoqiang Liu, Ju Guo, Luyao Chen, Gongxian Wang, Bin Fu

**Affiliations:** ^1^Department of Urology, The First Affiliated Hospital of Nanchang University, Yongwai Street 17, Nanchang, Jiangxi, China; ^2^Department of Urology, The First Hospital of Putian City, Putian, Fujian, China; ^3^Department of Urology, Shangrao Municipal Hospital, Shangrao, Jiangxi, China

## Abstract

**Background:**

Our team had firstly applied the transvesical approach to robot-assisted radical prostatectomy (RARP) in patients afflicted with localized prostate cancer (PCa). The present study aims to present the postoperative recovery of urinary continence (UC) following the anterior, transvesical, and posterior approaches to RARP for localized PCa and evaluate the independent predictors to early UC recovery after RARP.

**Methods:**

Patients harboring localized PCa and receiving anterior, transvesical, and posterior approaches to RARP between January 2017 and June 2020 were enrolled in this analysis. Results on UC recovery were compared between these three approaches with the Kaplan–Meier method. All clinical and pathological variables were further analyzed via univariable and multivariable regression analysis to determine the independent factors contributing to short-term UC recovery after RARP.

**Results:**

A total of 135, 73, and 66 instances were included in the anterior, transvesical, and posterior groups, respectively. Over the postoperative follow-up period, both the transvesical and posterior approaches showed an advantage over the anterior approach in promoting postoperative UC recovery (both *p* values <0.001). Three months after surgery, 55 (40.7%), 4 (5.5%), and 5 (7.6%) patients failed to UC in the anterior, transvesical, and posterior groups, respectively. Patient age, preoperative PSA, prostate volume, biopsy Gleason score, surgical approach, extended lymph node dissection technique, nerve-sparing technique, and positive lymph node were related to UC status based on univariable analyses (*p* < 0.05). Multivariable analysis results point patient age, prostate volume, surgical approach, and nerve-sparing technique as independent factors that affect postoperative UC recovery after RARP.

**Conclusions:**

The application of transvesical approach to RARP for localized PCa could obtain promising outcomes in terms of postoperative UC recovery. In addition, surgical strategies encompassing the nerve-sparing technique and the Retzius-sparing procedures, namely, the transvesical or posterior approach, during RARP could independently enable early achievement of postoperative continence.

## 1. Introduction

Postprostatectomy incontinence (PPI), an exceedingly adverse side effect of radical prostatectomy (RP), substantially decreases postoperative quality of life [1]. Despite the improvements in the knowledge of prostate anatomy and peripheral structures and the wide application of robot-assisted RP (RARP) [2, 3], the early return to urinary continence (UC) after RARP remains prolonged [2]. Considering the various definition of continence and surgeons experience, the reported PPI rates at 3 months after surgery range from 14% to 74% in series including >100 patients undergoing RARP [4] and the probabilities of PPI could reach up to 59% even in experienced hands at 3 months after surgery [4, 5], thus posing a considerable procedural limitation.

Several nonanatomic and surgical elements had been considered to affect the occurrence of PPI [6]. Thus, several surgical innovations have been developed to enhance the probability of early UC recovery [2, 7]. The Retzius-sparing/posterior approach to RARP, a technique preserving related anatomical structures in Retzius space [8], exhibits improved short-term continence rate. However, urologists were slow to adopt the posterior approach because of the steep learning curve and specific comments regarding the uncertainties of increased positive surgical margin (PSM) [9]. Recently, our team first conducted the transvesical approach to RARP, another procedure that avoids entry to the Retzius space, in patients afflicted with localized prostate cancer (PCa) [9–11], thus promoting early UC recovery. Based on the outcomes of transvesical RARP for localized PCa [9–11], the transvesical approach could serve as a valid alternative to RARP in selected patients, providing promising postoperative UC with compromising oncologic control for localized PCa.

Therefore, along with the perioperative, pathological, and urinary functional data pertaining to the consecutive patients receiving transvesical approach to RARP, we sought to present the UC outcomes following the anterior, transvesical, and posterior approaches to RARP for localized PCa and further identify the independent predictors to early UC status within 3 months after RARP.

## 2. Materials and Methods

The present study was retrospectively conducted upon the approval of the Institutional Review Board and Ethnic Committee of the First Affiliated Hospital of Nanchang University. Demographic, clinical, and pathologic data concerning patients with localized PCa undergoing the anterior, transvesical, or posterior approach to RARP between January 2017 and July 2020 were aggregated using our prospectively maintained database. Patients were enrolled into the present analysis under the following inclusion criteria: [1] patients undergoing RARP for localized PCa; [2] diseases in clinical T1-2 stage; and [3] absence of any clinical evidence of lymph node involvement or metastatic lesions. Patients with contraindications for RARP, neo-adjuvant hormone therapy, or suspected extracapsular extension in preoperative evaluation were excluded. When these conditions were simultaneously fulfilled, the instances were included in the final comparison. Preoperative assessment including prostate magnetic resonance imaging, bone scintigraphy, and abdominal computed tomography were routinely carried out in all cases.

**Figure 1:**
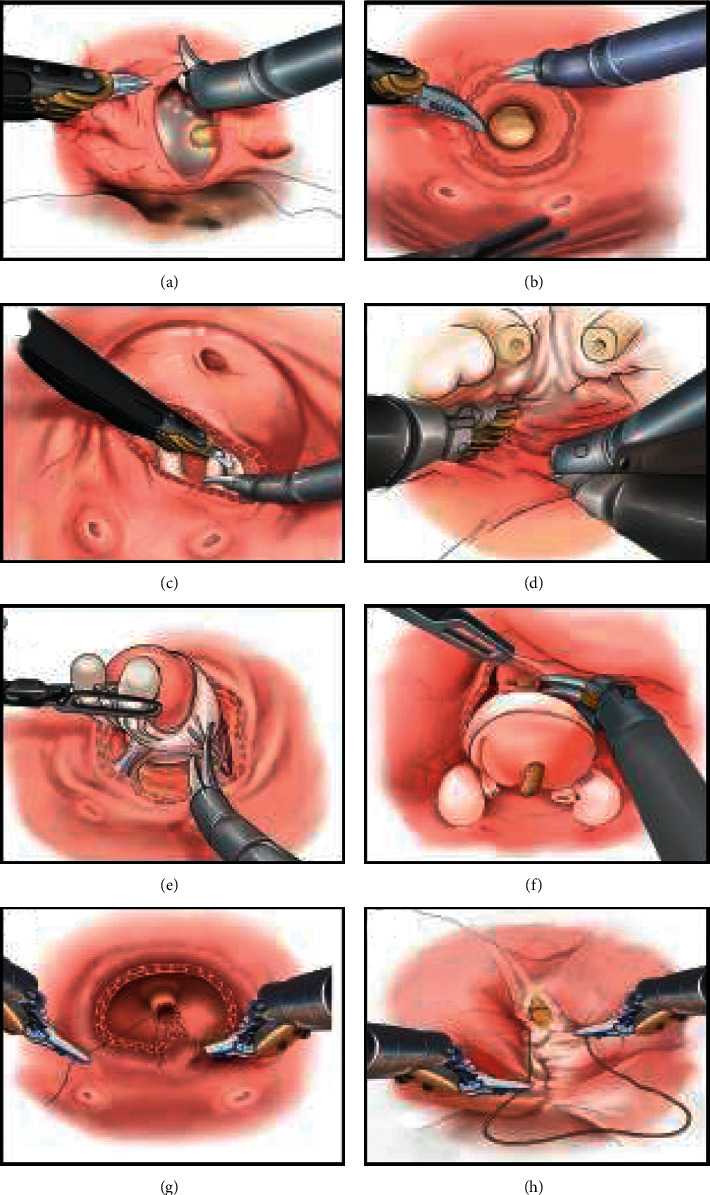
Surgical steps of transvesical robot-assisted radical prostatectomy. Through a vertical cystotomy (a), a circumferential incision was made around the internal urethral orifice (b). Dissections of the vas deferens and seminal vesicles were done through the lower half of the circumferential incision (c). Intrafascial posterior dissection was continued towards the apex (d). Lateral dissection of prostatic pedicles and neurovascular bundles was completed between the prostatic capsule and periprostatic fascia in a nerve-sparing manner (e). Anterior dissection continued towards the apex and urethra was exposed and transected (f). Urethrovesical anastomosis was achieved using two 4-0 barbed polydioxanone sutures on RB-1 needles in a running fashion (g). The bladder was closed in two layers in a running fashion (h).

All operations were completed by two highly experienced hands (Fu B and Wang GX), who had adopted standardized training in robotic surgery and performed over 300 RARPs before the initiation of study periods. The patients were assigned into the anterior and posterior groups at the discretion of these two surgeons according to the preoperative evaluation and patients' characteristics, while patients in the transvesical group were discretionarily enrolled after full comprehensions of why and how to perform the transvesical approach to RARP, the discrepancies between various approaches to RARP, and alternative therapies for cancer management. Then, the patients were provided with written informed consent with all details mentioned above. Written informed consent was obtained from each patient before launching the operations. The anterior approach was done following the modified technique proposed by Menon et al. [12], while the posterior approach was carried out as described by Galfano et al. [8]. As presented in our published studies [9–11], the detailed surgical steps of the transvesical approach to RARP are shown in Figure 1. Anatomically extended pelvic lymph node dissection (ePLND) was conventionally executed on condition that the preoperative estimated risk in lymph node metastasis exceeded 5%, while the performance of ePLND was routinely abandoned in patients with a lower risk of nodal involvement. A standardized ePLND template, including the removal of nodes overlying the external iliac artery and vein, nodes within the obturator fossa, nodes medial and lateral to the internal iliac artery, and nodes overlying the common iliac artery and vein up to the ureteral crossing, was utilized in all cases receiving lymph node dissections. The nerve-sparing technique was preoperatively scheduled depending on patients' clinical characteristics and intraoperatively adjusted based on the evidence of bundle involvement.

All information with regard to demographic variables covering age, body mass index (BMI), diabetes mellitus, hypertension, American Society of Anesthesiologists (ASA) score, and preoperative clinical tumor variables including preoperative total prostate specific antigen (PSA), clinical TNM stage, biopsy Gleason score, and prostate volume calculated using transrectal ultrasound were extracted from the database.

Information with respect to perioperative results incorporating operative time (OT), estimated blood loss (EBL), ePLND, nerve-sparing technique, open conversion, and transfusion and pathological outcomes (e.g., pathologic T stage, specimen Gleason score, PSM, and positive lymph node) was also retrieved from our database.

All patients were followed up for at least 12 months after surgery to evaluate postoperative UC recovery. The postoperative follow-ups were regularly arranged every 3 months within the first year after surgery and every 6 months since the second year after surgery. Other methods, such as outpatient visits and telephone interviews, were also carried out to gather postoperative outcomes about UC recovery. UC was defined as the prophylactic use of one dry pad or the absence of any pad within 24 h, and the results on the proportion of UC recovery were compared at the removal of catheter and at 3 and 12 months after surgery among these three surgical approaches.

Means and standard deviations were determined for the normally distributed continuous variables, while those with nonnormal distribution were presented as median and interquartile range. The Kruskal–Wallis test was employed to analyze the continuous variables between the three groups. All categorical variables were expressed as frequencies and proportions and compared with the Chi-square test. The proportions of UC recovery were compared between the three groups by using the Kaplan–Meier method. Univariable regression analyses were used to assess the effects of patient-related, pathologic, and technical factors on bivariate endpoints (incontinence vs. continence) at 3 months after surgery. Odds ratio and 95% confidence interval were determined. Variables with association (*p* value <0.10) in the univariate analyses were further evaluated in multivariable regression analyses. The STATA version 12.0 (STATA corp., College Station, TX) was used to conduct all statistical analyses with a two-sided *p* value <0.05 denoting statistical significance.

## 3. Results

Over the study period, based on the eligibility criteria, 274 patients experiencing RARP for localized PCa were included. A total of 135, 73, and 66 men underwent the anterior, transvesical, and posterior approaches to RARP, respectively. Significant differences were observed in the baseline features between these three groups (Table 1). The anterior group was related to a higher mean age, lower mean BMI, higher mean preoperative total PSA, and larger mean prostate volume than the transvesical group, while the rate of diabetes mellitus (25.2%) in the anterior group was higher than that in the transvesical (13.7%) and posterior (12.1%) groups. The proportions of ASA score (≥3) and lesions in clinical T1c stage in the anterior group were higher than those in transvesical and posterior groups. No significant differences were observed in the rate of hypertension between the three arms (*p*=0.323). Men receiving the anterior approach to RARP had higher median specimen Gleason score than those undergoing the transvesical or posterior approach to RARP.

Perioperative and pathologic outcomes are presented in Table 2. All operations were successfully completed without open conversion in all groups. The mean OT in the anterior group was lower than that in the transvesical and posterior groups, whereas no significant difference was observed in the mean EBL between the three groups (*p*=0.247). ePLND was performed in 37 (27.4%), 8 (11.0%), and 6 (9.1%) instances with *p*=0.001, while lymph node invasion was detected in 12 (8.9%), 3 (4.1%), and 3 (4.5%) cases in the anterior, transvesical, and posterior groups, respectively (*p*=0.389). The nerve-sparing technique was applied in 92 (68.1%), 69 (94.5%), and 61 (92.4%) patients in the anterior, transvesical and posterior groups, respectively (*p*=0.001). Patients in these three groups had comparable proportions of transfusion and PSM (*p*=0.561 and *p*=0.637, respectively). The anterior group had a tendency towards a lower rate of pT2 diseases than the two other groups, while the median specimen Gleason score in the transvesical group was lower than that in the anterior group.

**Figure 2:**
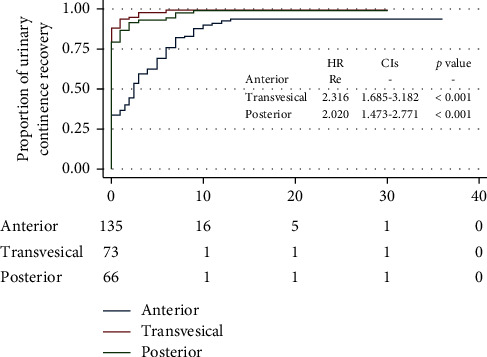
Kaplan–Meier curves showing the proportion of urinary continence (UC) in patients undergoing the anterior, transvesical, and posterior approaches to robot-assisted radical prostatectomy during the follow-up intervals. UC was defined as requiring no pad or preventively using one dry pad per day.

The Foley catheter was routinely removed at two weeks after surgery in the anterior group, while the removal of Foley catheter was usually done at one week after surgery in the transvesical and posterior groups.  Table 3 delineates the proportions of continence recovery at different postoperative time points. The percentages of patients achieving UC recovery in the anterior group were lower than that in the transvesical and posterior groups at the removal of the catheter, at 3, 6, and 12 months after surgery. The Kaplan–Meier curves illustrated that the accumulative likelihood of postoperative UC recovery in the anterior group was significantly lower than that in the transvesical arm (*p* < 0.001) and posterior arm (*p*=0.001) over the whole follow-up periods (Figure 2).

The results of univariable and multivariable regression analyses of the predictors of urinary incontinence at postoperative 3 months are displayed in Table 4. Based on the univariable analyses, patients aged >65 years were at higher risks of early incontinence than younger patients (OR: 1.308, 95% CIs: 1.036–1.711, *p*=0.022). Preoperative total PSA >20 ng/ml compared with ≤20 ng/ml was also a risk factor for the decreased UC rate (OR: 1.124, CIs: 1.003–3.155, *p*=0.042). Larger prostate volume, higher clinical TNM stage, and positive lymph node were significantly related to postoperative return to UC. Intriguingly, the applications of nerve-sparing technique, ePLND, and the transvesical or posterior approach also had significant correlations with the achievement of early UC (all *p* values <0.05). Based on multivariable regression analyses, patient age >65 versus ≤65 years and prostate volume >40 versus ≤40 ml were detected as independent contributing factors to postoperative incontinence (*p*=0.015 and *p*=0.031, respectively), while the performances of nerve-sparing technique and transvesical and posterior approach enhanced the early recovery of postoperative UC (*p*=0.022, *p* < 0.001, and *p* < 0.001, respectively).

## 4. Discussion

The appearance of urinary incontinence after RP can significantly reduce the postoperative quality of life [2, 13]. Despite the advantages provided by robotic technology, including three-dimensional visualization, wristed instrumentation, and magnification, the incidence of urinary incontinence after RARP during the early postoperative period remained relatively high [2]. In the present study, we explored 19 parameters of early UC recovery at 3 months after surgery in 274 men receiving RARP for managing localized PCa. Interestingly, our results demonstrated that young patient age, small prostate volume, nerve-sparing technique, and transvesical or posterior approach were independent driving factors for early urinary function recovery.

Young age was significantly associated with a high possibility of early UC recovery within 3 months after surgery, which may be attributable to the fact that the rate of preexisting lower urinary tract symptoms caused by an enlarging prostate and/or age-related functional changes in the urinary bladder and urethra in older patients was higher than that in younger patients [6]. In concurrence with the results of our study, Novara et al. [14] discovered that patients who recovered continence at postoperative 12 months were significantly younger than those in the incontinent group involving 308 men receiving RARP. Palisaar et al. [7] also reported that age is an important predictor of urinary functional outcomes based on a retrospective study reviewing 4,028 consecutive patients undergoing RP. Matsushita et al. [15] also found that young age is independently related to the likelihood of continence recovery at 6 and 12 months after prostatectomy in an analysis including 2,849 PCa patients.

Consistent with other reported data, our results revealed that larger prostate volume could negatively affect the early achievement of UC within 3 months after surgery in an independent manner. Although the improved visualization and increased precision offered by the robotic platform can remarkably lessen the operative invasiveness and reduce the risk of organ injuries when performing RARP, the resection of parts of the urethra when removing a larger prostate is relatively longer than that in patients with a smaller prostate, resulting in worse continence outcomes [6, 16, 17]. Furthermore, postoperative incontinence could be partly explained by the high incidences of preexisting lower urinary tract symptoms among patients with a large prostate [6, 18]. In a retrospective analysis incorporating 355 patients receiving RARP, Boczko et al. [19] found that the postoperative 6-month continence rate among patients with a prostate size >75 cm^3^ was significantly lower than that among men with a prostate size <75 cm^3^. Konety et al. [20] also found that cases with >50 cm^3^ prostate size had lower rates of continence at postoperative 6 and 12 months after RP than those with ≤50 cm^3^ in an analysis with 2,097 patients.

In the present study, the application of nerve-sparing technique enhanced the early UC recovery after RARP. A careful execution in preparing the surrounding structures and better preservation of anatomic integrity and innervation of the sphincter complex during the nerve-sparing procedure may help in interpreting the promising UC recovery in instances receiving nerve-sparing technique. Srivastava et al. [21] demonstrated that patients with high degree of nerve preservation achieved superior returns of UC without compromising oncologic safety in 1,417 patients treated with RARP by a single surgeon. In concurrence with our results, the UC proportion (71.8%) in patients undergoing nerve-sparing technique was significantly higher than that in patients (43.5%) without nerve preservation at postoperative 3 months, demonstrating the crucial effects of nerve-sparing technique on postoperative UC recovery in patients undergoing RARP.

Based on our results, both the transvesical and posterior approaches are superior over the anterior approach concerning postoperative early return to UC, which could be attributed to the common advantage of these two Retzius-sparing surgeries. Both the transvesical and posterior techniques allowed the prostate gland to be removed without disrupting the integrity of Retzius space, thus providing a strong rationale for achieving enhanced UC recovery after RARP [9, 11]. All UC-related structures in the Retzius space, such as the endopelvic fascia, puboprostatic ligaments, and detrusor apron, were preserved to provide a strong supportive mechanism and stabilize the urethra [2, 22, 23]. Unlike the posterior approach, the transvesical method, which was firstly applied by our team, was carried out similarly to the transperitoneal anterior technique after bladder neck excision with Douglas' pouch being preserved [10, 24]. In the present study, the continence rates at postoperative 3 months in both the transvesical (94.5%) and posterior (92.4%) groups were comparable with various studies testing the posterior approach, with rates of 59.7%–94.9% [25–27], while the continence rate (59.3%) obtained after the anterior approach was similar to that (29.5%–73.7%) acquired using the anterior method in reported data [28]. Based on the multivariable analyses, our study revealed the Retzius-sparing technique, namely, the transvesical and posterior approach, could independently account for early UC achievement within postoperative 3 months.

Some limitations affect the generalizability of our results. Structural shortages are involved in data collection because of the retrospective nature of this analysis. Moreover, the clinical endpoint, UC, was regarded as no or one pad usage per day, which may be imprecise but reflects prior practice and therefore guarantees the similarity across different studies. Furthermore, the study population was relatively small in three groups of this study. In addition, preexisting lower urinary tract symptoms that could potentially affect continence status were not recorded in our prospectively maintained database.

Notwithstanding these limitations mentioned above, this study was the first to evaluate the continence status acquired after the anterior, transvesical, and posterior approaches to RARP for localized PCa. In combination with the outcomes of transvesical RARP, we further analyzed the independent factors associated with early postoperative UC recovery at postoperative 3 months. Our study represents a natural process during the development of a newly applied surgical technique, and the conclusions were drawn and strengthened on the basis of rigorous methodology.

## 5. Conclusions

The application of transvesical approach to RARP for localized PCa could obtain promising outcomes in terms of postoperative UC recovery. In addition, surgical strategies encompassing the nerve-sparing technique and the Retzius-sparing procedures, namely, the transvesical or posterior approach, during RARP could independently enable the early achievement of postoperative continence. This conclusions needs to be further validated in well-designed prospectively randomized trials with large sample sizes.

## Figures and Tables

**Table 1:** Demographic and clinical characteristics of the analytic cohort.

Variables	Anterior approach (*n* = 135)	Transvesical approach (*n* = 73)	Posterior approach (*n* = 66)	*p* ^†^
Age, years, mean (SD)	67.6 (7.2)	63.4 (7.1)	66.5 (7.3)	0.001
BMI, kg/m^2^, mean (SD)	22.6 (3.7)	23.7 (3.8)	25.1 (4.5)	0.001
Diabetes mellitus (yes), *n* (%)	34 (25.2%)	10 (13.7%)	8 (12.1%)	0.035
Hypertension (yes), *n* (%)	41 (30.4%)	26 (35.6%)	27 (40.9%)	0.323
ASA score (≥3), *n* (%)	13 (9.6%)	5 (6.8%)	4 (6.1%)	0.621
Preoperative total PSA, ng/mL, mean (SD)	24.7 (12.4)	19.8 (6.1)	17.8 (6.7)	0.001
Prostate volume, mL, mean (SD)	42.7 (13.7)	36.8 (9.6)	38.7 (14.2)	0.004
Clinical TNM stage, *n* (%)				0.002
T1c	59 (43.7%)	28 (38.4%)	16 (24.2%)	
T2a-b	55 (40.7%)	42 (57.5%)	44 (66.7%)	
T2c	21 (15.6%)	3 (4.1%)	6 (9.1%)	
Biopsy Gleason score, median (IQR)	7 (6.8)	6 (5.7)	6 (5.7)	0.001

SD: standard deviation; BMI: body mass index; ASA: American Society of Anesthesiologists; IQR: interquartile range. ^†^Continuous variables were compared using the Kruskal–Wallis test, and categorical variables were compared using the Chi-square test.

**Table 2:** Perioperative and pathologic outcomes divided by surgical approaches.

Variables	Anterior approach (*n* = 135)	Transvesical approach (*n* = 73)	Posterior approach (*n* = 66)	*p* ^†^
Operative time, min, mean (SD)	117.7 (25.0)	133.3 (27.7)	128.4 (29.0)	0.001
Estimated blood loss, mL, mean (SD)	98.6 (48.5)	111.9 (62.8)	105.5 (75.7)	0.247
ePLND, *n* (%)	37 (27.4%)	8 (11.0%)	6 (9.1%)	0.001
Open conversion, *n* (%)	0 (0%)	0 (0%)	0 (0%)	—
Transfusion, *n* (%)	5 (3.7%)	1 (1.4%)	3 (4.5%)	0.561
Nerve-sparing technique, *n* (%)	92 (68.1%)	69 (94.5%)	61 (92.4%)	0.001
Postoperative pathology				
Pathological T stage, *n* (%)				0.001
pT2	91 (67.4%)	63 (86.3%)	59 (89.4%)	
pT3	44 (32.6%)	10 (13.7%)	7 (10.6%)	
Specimen Gleason score, median (IQR)	7 (5.8)	6 (5.7)	7 (5.7)	0.038
Positive surgical margin, *n* (%)	25 (18.5%)	11 (15.1%)	9 (13.6%)	0.637
Positive lymph node, *n* (%)	12 (8.9%)	3 (4.1%)	3 (4.5%)	0.389

ePLND: extended pelvic lymph nodes dissection; SD: standard deviation; IQR: interquartile range. ^†^Continuous variables were compared using the Kruskal–Wallis test, and categorical variables were compared using the Chi-square test.

**Table 3:** Perioperative urinary continence recovery divided by surgical approaches.

Urinary continence	Anterior (*n* = 135)	Transvesical (*n* = 73)	Posterior (*n* = 66)	*p* value^†^
Continence on removal of the catheter, *n* (%)	46 (34.1%)	64 (87.7%)	52 (78.8%)	<0.001
Continence at postoperative 3 months, *n* (%)	80 (59.3%)	69 (94.5%)	61 (92.4%)	<0.001
Continence at postoperative 6 months, *n* (%)	102 (75.6%)	73 (100.0%)	62 (93.9%)	<0.001
Continence at postoperative 12 months, *n* (%)	123 (91.1%)	73 (100.0%)	66 (100.0%)	0.001

^†^Categorical variables were compared using the Chi-square test.

**Table 4:** Univariable and multivariable cox proportional hazards regression analysis: factors associated with short-term continence recovery.

Variables	Univariable analysis			Multivariable analysis		
	OR	95% confidence intervals	*p* value	OR	95% confidence intervals	*p* value
Age (years)						
≤65	Ref	—	—	Ref	—	—
>65	1.308	1.036–1.711	0.022	1.541	1.128–1.974	0.015
BMI (kg/m^2^)						
≤23	Ref	—	—			
>23	0.676	0.410–1.116	0.126			
Diabetes mellitus						
No	Ref	—	—			
Yes	0.893	0.534–1.221	0.412			
Hypertension						
No	Ref	—	—			
Yes	1.012	0.762–1.516	0.675			
ASA score						
<3	Ref	—	—			
≥3	1.503	0.912–2.476	0.110			
Preoperative total PSA (ng/mL)						
≤20	Ref	—	—	Ref	—	—
>20	1.124	1.003–3.155	0.042	1.058	0.842–2.155	0.089
Prostate volume (mL)						
≤40	Ref	—	—	Ref	—	—
>40	1.587	1.368–1.872	0.025	1.558	1.281–1.119	0.031
Clinical TNM stage						
T1c	Ref	—	—	Ref	—	—
T2a-b	1.323	0.952–1.821	0.075	1.583	0.313–2.186	0.118
T2c	1.761	0.833–3.121	0.535	1.617	0.467–2.513	0.321
Biopsy Gleason score						
≤6	Ref	—	—	Ref	—	—
=7	0.774	0.422–1.419	0.407	0.804	0.519–1.927	0.539
≥8	1.848	1.006–3.397	0.048	1.628	0.835–2.393	0.108
Surgical approach						
Anterior approach	Ref	—	—	Ref	—	—
Transvesical approach	0.055	0.013–0.227	<0.001	0.048	0.012–0.199	<0.001
Posterior approach	0.156	0.062–0.391	<0.001	0.151	0.059–0.377	<0.001
Operative time (min)						
≤120	Ref	—	—			
>120	0.669	0.401–1.116	0.124			
Estimated blood loss (ml)						
≤100	Ref	—	—			
>100	1.030	0.605–1.754	0.913			
ePLND						
No	Ref	—	—	Ref	—	—
Yes	1.753	1.015–2.921	0.031	1.448	0.993–2.115	0.097
Transfusion						
No	Ref	—	—			
Yes	1.468	0.646–3.452	0.462			
Nerve-sparing technique						
No	Ref	—	—	Ref	—	—
Yes	0.312	0.125–0.754	0.013	0.327	0.159–0.876	0.022
Pathological T stage						
pT2	Ref	—	—			
pT3	0.935	0.425–2.413	0.522			
Specimen Gleason score						
≤6	Ref	—	—			
=7	0.613	0.332–1.135	0.119			
≥8	0.686	0.350–1.345	0.272			
Positive surgical margin						
No	Ref	—	—	Ref	—	—
Yes	0.896	0.484–1.003	0.060	0.957	0.436–1.073	0.091
Positive lymph node						
No	Ref	—	—	Ref	—	—
Yes	0.833	0.585–0.995	0.047	0.923	0.741–1.611	0.101

OR: odds ratio; BMI: body mass index; ASA: American Society of Anesthesiologists; ePLND: extended pelvic lymph nodes dissection.

## Data Availability

The datasets generated for this study are available upon request to the corresponding author.
